# When Social Isolation Might Save Your Life: An MIT AgeLab Study of Living Through the COVID-19 Crisis at Age 85 and Over

**DOI:** 10.1093/geroni/igaa057.1560

**Published:** 2020-12-16

**Authors:** Julie Miller, Taylor Patskanick, Lisa D’Ambrosio, Martina Raue, Alexa Balmuth, Joseph Coughlin

**Affiliations:** 1 MIT AgeLab, Cambridge, Massachusetts, United States; 2 MIT, Cambridge, Massachusetts, United States; 3 Massachusetts Institute of Technology, Cambridge, Massachusetts, United States

## Abstract

Previous research shows that social isolation is associated with increased mortality rates among older adults. However, in the face of the novel coronavirus (COVID-19), social distancing was prescribed as a potentially lifesaving measure, particularly for older adults. In this session, MIT AgeLab researchers will present findings from a mixed methods study with the MIT AgeLab 85+ Lifestyle Leaders, a panel study of octogenarians and nonagenarians that began in September 2015. In March 2020, AgeLab researchers began conducting a series of telephone interviews (n=15) with Lifestyle Leaders and collected quantitative data via an online survey (n=25). Together, the interviews and survey inquired about Lifestyle Leaders’ attitudes and behaviors related to COVID-19, as well as their uses of technology throughout the crisis. Findings suggest that fears about the impacts of coronavirus transcended multiple domains of the Lifestyle Leaders’ lives, including their physical, socioemotional, and financial wellbeing. Lifestyle Leaders were asked about functional and emotional repercussions of social distancing and other precautions they had taken to reduce the spread of COVID-19 and to protect themselves from contracting the virus. Special attention was paid to differences in lived experiences of the COVID-19 crisis among Lifestyle Leaders who were living independently versus those who were living in assisted living or continuing care retirement communities. Findings from this study suggest that socially-connected emergency preparedness measures will become increasingly important for the growing number of octogenarians and nonagenarians.

